# Food habits in pregnancy and its association with gestational diabetes mellitus: results from a prospective cohort study in public hospitals of urban India

**DOI:** 10.1186/s40795-020-00388-x

**Published:** 2020-11-19

**Authors:** R. Deepa, Melissa Glenda Lewis, Onno C. P. Van Schayck, Giridhara R. Babu

**Affiliations:** 1 Indian Institute of Public Health-Bangalore, Public Health Foundation of India (PHFI), Bangalore, India; 2 Indian Institute of Public Health-Hyderabad, Public Health Foundation of India (PHFI), Hyderabad, India; 3 Care and Public Health Research Institute, Maastricht University, Maastricht, Limburg The Netherlands; 4 Lifecourse epidemiology, Indian Institute of Public Health-Bangalore, Public Health Foundation of India (PHFI), Magadi Road I cross, SIHFW premises, Bengaluru, 560023 India; 5 Public health and clinical medicine, Wellcome Trust/DBT India Alliance, New Delhi, India

**Keywords:** Gestational diabetes, Diet, Red meat, Pregnancy, India

## Abstract

**Background:**

Few studies have explored the relationship between food habits and the risk of gestational diabetes mellitus (GDM) in women from India. We aimed to investigate the associations of food habits and the risk of GDM.

**Methods:**

As part of the MAASTHI prospective cohort study in urban Bengaluru, India, pregnant women between 18 and 45 years, less than 36 weeks of gestation were included. During baseline, the participant’s age, education, physical activity levels, and food habits were recorded. Screening of GDM was done by the World Health Organization diagnostic criteria using a 2-h 75-g oral glucose tolerance test between the 24th–36th weeks of gestation.

**Results:**

We included 1777 pregnant women in the study. We show that 17.6% of the women had GDM, of which 76.7% consume red meat. Red meat consumption was associated with an increased risk of GDM (aRR = 2.1, 95% CI 1.5, 2.9) after adjusting for age, family history of diabetes and socioeconomic status.

**Conclusion:**

The high intake of red meat consumption in pregnancy needs further examination. Also, future evaluations should consider evaluating the risk of red meat consumption against the combined effect of inadequate consumption of vegetables, fruits, and dairy products in pregnant women. Interventions to educate women in lower socioeconomic status on inexpensive, seasonal, and healthy food might be helpful.

## Background

Gestational diabetes mellitus (GDM) is carbohydrate intolerance resulting in hyperglycemia of variable severity with onset or first recognition during pregnancy [1]. GDM can lead to several adverse outcomes in the infant. These include fetal hyperglycemia, macrosomia, shoulder dystocia, respiratory distress syndrome, fetal hypoglycemia, prematurity, hypocalcaemia, and hyperbilirubinemia [2–6]. Additionally, children borne of women with GDM are more likely to develop obesity and type 2 diabetes mellitus (T2DM) [7, 8]. Also, the mother is at risk for preeclampsia, caesarean sections, and increased risk in the future for T2DM. Globally, GDM affects 18.4 million women, which accounts for 86.4% of gestational hyperglycemia, defined as any higher levels of glucose [9]. In South Asia alone, the prevalence of GDM is increasing with current estimates indicating 26.6% [9] and in urban India vary in different regions, from 0.56 to 41.9% [10, 11]. Therefore, early detection and treatment of GDM must be implemented to improve outcomes for both mother and child in these women [12, 13].

Advanced maternal age, high body mass index (BMI), a family history of T2DM are significant independent risk factors for hyperglycemia in pregnancy [14]. Among other modifiable risk factors of GDM, tackling obesity is essential; even moderate changes in pre-pregnancy weight have shown to affect the risk of gestational diabetes among obese women [5, 15–17]. Studies have also shown that minor degrees of carbohydrate intolerance are related to obesity [18]. Furthermore, Indians are known to have the thin-fat phenotype that is characterized by an excessive accumulation of fat for lower BMI [19]. Several studies have proven that adults who had a low birth weight are more likely to develop higher glucose concentration and insulin resistance and adiposity [20]. Low birth weight and subsequent risk of non-communicable diseases have been associated with maternal diets that are poor in micronutrients [21]. Therefore lifestyle factors such as lack of exercise and diet composition are modifiable predictors of risk for abnormal glucose tolerance during pregnancy. Hence apart from screening and managing GDM, it is essential to identify and control the factors leading to glucose intolerance in pregnancy. In the Indian context, identifying nutritional risk factors will enable us to screen and prevent GDM in this setting.

The available evidence regarding the relation of GDM with nutritional factors is not clear. Some studies have suggested that diets high in total fat, saturated fat, and lower consumption of carbohydrate, fruits, and vegetables during pregnancy are associated with a higher risk of developing GDM [22–25]. The SUN cohort in Spain demonstrates high consumption of potato, fast food, and sugar-sweetened beverages before pregnancy are independently associated with GDM [26, 27]. Studies that have examined food groups and dietary patterns found that GDM was predicted by high intake of red and processed meats and a Western-type dietary pattern (i.e., high in red meat, refined sugars, and fried or snack foods) [28, 29].

Few studies have explored the relationship between dietary patterns and the risk of GDM of women from India, especially from the lower socioeconomic settings. There is limited knowledge regarding dietary habits as well as its association with GDM in pregnant Indian women attending public hospitals. As a result, there is a coexistence of malnutrition in the mother and the fetus associated with these conditions [30]. Understanding the nutritional risk factors and its association with GDM can inform the policies in improving the health of women attending public hospitals. We aimed to investigate associations of food habits in pregnant women with the risk of GDM in the urban Indian women of the lower socioeconomic strata. We hypothesize that low intake of vegetables and high animal origin food among pregnant women of lower socioeconomic strata are associated with the risk of GDM.

## Methods

### Study design and setting

Maternal Antecedents of Adiposity and Studying the Transgenerational role of Hyperglycemia and Insulin (MAASTHI) is an ongoing prospective cohort study. The cohort recruited pregnant women from public hospitals in Bengaluru, India. The primary objective of the MAASTHI Cohort is to study the association between maternal hyperglycemia and the risk of non-communicable diseases in infants. The participating hospital had an obstetric department that caters to the urban population of Bengaluru in the surrounding vicinity. A detailed protocol and related publications can be found elsewhere [17, 31].

In brief, we screened all pregnant women attending antenatal clinics in public hospital and obtained consent from eligible women to participate voluntarily in the cohort. The participants were enrolled from April 2016 to September 2018. Women entered the study if a singleton pregnancy of 14-weeks’ gestation was confirmed. All pregnant women between the age of 18–45 years less than 36 weeks of gestation and willing to provide informed consent were included. We excluded women with coexisting severe illness. Ultrasonography reports were used to estimate the gestational age, and in the absence of ultrasonography reports, the participant’s last menstrual period was recorded.

### Food habit assessment

The food habits of the participants were recorded at the baseline using an interviewer-administered food propensity questionnaire to elicit the frequency of food consumption in the last month. Based on the cultural and local context, we prepared a list of foods more frequently consumed in this population. Food groups like vegetables, fruits, eggs, dairy, red meat, chicken, fish, nuts, cereal, cooking oil, fried food, and other habits like eating out were recorded. The participants were asked to indicate how often they consumed the particular food item. The questionnaire had five options for frequency of consumption ranging from “Never” to “daily.” (Additional file 1). The questionnaire was administered before undergoing the oral glucose tolerance test.

### Assessment of non-dietary covariates

After the baseline interview, our trained research staff recorded the anthropometric measurements in a separate room, assuring privacy to the pregnant women. Using a digital weighing scale (Tanita) weight to the nearest 0.1 kg and using portable stadiometer (SECA 213) height of the mother to the nearest 0.1 cm were measured. The skinfold thickness at biceps, triceps and subscapular sites were measured using a Holtain skinfold caliper; (Holtain 610ND) and the average of three readings was taken. Sum of skinfold was calculated by adding bicep, triceps and subscapular skinfolds. We assessed the physical activity levels of the pregnant woman using a validated questionnaire [32] that captures the activities performed in a week, the duration of each activity, and frequencies of the activities were noted. We estimated the Metabolic Equivalent of Task (MET) values of each activity by multiplying MET allotted value, duration of the activity, and frequency performed in a week. The level of physical activity was defined as low if it was < 600 METs, moderate when 600–2999 METs, and as high when it is (≥3000 METs. The socioeconomic status was categorized based on the Kuppuswamy scale using education, occupation, and income, into upper, upper- middle, lower-middle, upper-lower, and lower. For analysis, purpose only two main categories of lower (upper-lower, and lower) and middle (upper middle, lower middle) was created [33].

### Ascertainment of GDM

A 2-h 75-g oral glucose tolerance test was performed during the 24th–36th week of gestation. Diagnostic cut-off points, as defined by the International Association of the Diabetes and Pregnancy Study Groups (IADPSG), for fasting ≥92 mg/dL and 2-h (postprandial) ≥153 mg/dL. One glucose value equal to or above any cut-off point was considered for the diagnosis of GDM [34]. Pregnant women’s hemoglobin values were also tested, and values less than 11 g/dL was considered as anemia.

### Statistical analyses

Descriptive statistics were performed using IBM SPSS Software Version 23 [35]. It was done for maternal characteristics and food habits for women with and without GDM. Categorical variables were expressed as frequency and percentages (%) and normally distributed variables as mean and standard deviation. Missing data were analyzed using available case analysis. Association between food habits with GDM status was assessed using the Chi-square test to determine the food variables for multivariable regression models. Modified Poisson regression with robust variance analysis was performed in R Version 3.6.2 [36] to determine the association between food habits with GDM. This was adjusted for maternal age, family history of diabetes, socioeconomic class, body mass index (BMI), the sum of skinfold thickness, metabolic equivalent of task (MET) and gestational age. Confounders were selected based on the parameters that were significant in univariate analysis. Adjusted relative risk (aRR), unadjusted relative risk (RR), 95% confidence intervals (CI) along with *p*-values have been reported, where *p* values ≤0.05 is considered statistically significant.

## Results

A total of 1777 pregnant women from the ongoing MAASTHI cohort were selected for this study. The mean age was 24.3 years, 50.3% were Muslim, 96.9% attended school, and most of them were unemployed (92.5%). A majority (62.7%) belonged to lower socioeconomic status. Of the total, 45.0% of the women were nulliparous, 43.5% anemic, 22.1% had a history of abortion, and 17.6% (*n* = 313) were diagnosed with GDM in the current pregnancy.

The mean age of women with GDM (25.9 ± 4.4 years) was higher compared to women without GDM (24.0 ± 4.0 years) and was statistically significant. We found that socioeconomic status of women and having a family history of diabetes mellitus was statistically significant with GDM. Women with GDM had a higher sum of skinfold thickness (18.4 ± 4.7) compared to normoglycemic women (15.3 ± 4.6). Women diagnosed with GDM also had higher BMI (27.3 ± 5.2) when compared to women without GDM (24.4 ± 4.3). (Table 1).

**Table 1:** Distribution of demographic characteristics among pregnant women with GDM and without GDM (*N* = 1777)

Characteristics of the cohort	Categories	GDM status		Total	***P***-value
		Yes (***n*** = 313)	No (***n*** = 1464)	N (%)	
Age (years)	Mean ± SD	25.9 ± 4.4	24.0 ± 4.0	24.3 ± 4.1	< 0.001*
Gestational age at baseline (weeks)	Mean ± SD	23.9 ± 5.4	23.8 ± 5.4	23.8 ± 5.3	0.95
Gestational age at the time of OGTT weeks)	Mean ± SD	28.1 ± 2.6	28.1 ± 2.9	28.1 ± 2.9	0.90
Religion	Hinduism	142(45.4)	677(46.2)	819 (46.0)	0.95
	Christianity	12(3.6)	53(3.7)	65 (3.7)	
	Islam	159(50.8)	734(50.1)	893 (50.3)	
Participant Education	Illiterate	12(3.8)	43(2.9)	55(3.1)	0.06
	Primary School	21(6.7)	78(5.3)	99(5.6)	
	Middle School	43(13.7)	267(18.2)	310(17.4)	
	High School	134(42.8)	641(43.8)	775(43.6)	
	PUC or Diploma^a^	64(20.4)	309(21.1)	373(21.0)	
	Graduate and above	39(12.5)	126(8.6)	165(9.2)	
Husband’s Education	Illiterate	28(8.9)	139(9.5)	167(9.4)	0.41
	Primary School	27(8.6)	150(10.2)	177(10.0)	
	Middle School	57(18.2)	281(19.2)	338(19.0)	
	High School	124(39.6)	559(38.2)	683(38.4)	
	PUC or Diploma^a^	59(18.8)	214(14.6)	273(15.4)	
	Graduate and above	18(5.8)	115(7.9)	133(7.5)	
	Do not know	0(0.0)	6(0.4)	6(0.3)	
Participant’s Occupation^b^	Unemployed	283(90.4)	1361(93.0)	1644(92.5)	0.10
	Unskilled	15(4.8)	70(4.8)	85(4.8)	
	Semi-skilled/ Skilled	15(4.8)	33(2.3)	48(2.7)	
Husband’s Occupation^b^	Unemployed	1(0.3)	4(0.3)	5(0.3)	0.30
	Unskilled	155(49.5)	776(53.0)	931(52.4)	
	Semi-skilled	88(28.1)	405(27.7)	488(27.5)	
	Skilled	60(19.2)	223(15.2)	283(15.9)	
	Clerical or Professional	14(4.5)	56(3.8)	70(4.0)	
Socioeconomic class	Lower	177(56.5)	937(64.0)	1114 (62.7)	0.01*
	Middle	136(43.5)	527(36.0)	663 (37.3)	
Gravida	One	105(33.5)	588(40.2)	693(39.0)	0.10
	Two	129(41.2)	546(37.3)	675(38.0)	
	Three or more	79(25.2)	330(22.5)	409(23.0)	
Parity	Nulliparous	133(42.5)	666(45.5)	799 (45.0)	0.62
	Primiparous	143(45.7)	637(43.5)	780(43.9)	
	Multiparous	37(11.8)	161(11.0)	198(11.1)	
History of Abortion	No history	230(73.5)	1154(78.8)	1384(77.9)	0.07
	Once	68(21.7)	252(17.2)	320(18.0)	
	≥ twice	15(4.8)	58(4.0)	73(4.1)	
Anemia	Present	127(40.6)	645(44.1)	772(43.5)	0.24
	Absent	186(59.4)	815(55.7)	1001(56.3)	
Family history of Diabetes Mellitus	Absent	197(62.9)	1134(77.5)	1331(75.0)	< 0.001*
	One parent	92(29.4)	286(19.5)	378(21.3)	
	Two parents	24(7.7)	40(2.7)	64(3.6)	
Metabolic Equivalent of Task (MET)	Low	8(2.6)	35(2.4)	43(2.4)	0.8
	Moderate	305(97.4)	1429(97.6)	1734(97.6)	
Sum of skin fold thickness (mm)	Mean ± SD	18.4 ± 4.7	15.3 ± 4.6	15.9 ± 4.8	< 0.001^*^
Body mass index (BMI)	Mean ± SD	27.3 ± 5.2	24.4 ± 4.3	24.9 ± 4.6	< 0.001^*^

* Statistically significant at 5% level of significance

^a^*PUC* Pre-university certification

^b^Unskilled: laborer, construction laborer, helper, attender; peon, cleaner, sweeper

Semi-Skilled: Gatekeeper/Security, Asst. Operator, Asst. electrician, waiter

Skilled: Tailor, carpenter, driver, plumber, electrician

### Food habit and its association with GDM

Among women with GDM, 76.7% consume red meat, 66.3% consume fried food, 42.9% eat out, 98.8% eat fruits, 97.8% consume vegetables, 90.8% consume polished rice, only 8.0% use palm oil for cooking, 28.7% drink coffee, 50.0% drink tea, 83.4% consume dairy products, 76.1% consume eggs, 82.2% consume chicken, and 66.2% consume fish. However, there was no statistically significant difference between women with and without GDM, in most of the food types (Table 2). Fruits and vegetable consumption (> 6 servings) was higher in women with normal glucose levels compared to women with GDM (6.8% v/s 4.1%). We found that red meat (*p* value = 0.02) intake was significantly associated with GDM.

**Table 2:** Univariate analysis of dietary habits with GDM status of pregnant women (*N* = 1777)

Dietary habit	Categories	GDM status		Total	***P***-value
		Yes (***n*** = 313)	No (***n*** = 1464)	n(%)	
Red meat consumption	Never	73(23.2)	420(28.7)	493(27.7)	0.02^*^
	< 1 per month	13(4.1)	27(1.8)	40(2.3)	
	1–3 times /month	53(17.2)	190(12.9)	243(13.7)	
	1–3 times/week	153(48.7)	745(50.8)	898(50.5)	
	> 3 times / week	10(3.2)	36(2.4)	46(2.6)	
	Daily	11(3.5)	46(3.1)	57(3.2)	
Fried food	Never	105(33.8)	470 (32.1)	575(32.4)	0.96
	< 1 per week	93(29.6)	448(30.6)	541(30.4)	
	1–3 times a week	101(32.2)	470(32.1)	571(32.1)	
	4 to 6 times a week	5(1.6)	25(1.7)	30(1.7)	
	Daily	9(2.9)	51(3.4)	60(3.4)	
Eating out	Never	178(57.0)	821(56.0)	999(56.2)	0.96
	< 1 per week	87(27.7)	428(29.2)	515(29.0)	
	1–3 times a week	46(14.6)	205(14.0)	251(14.1)	
	4 to 6 times a week	1(0.3)	7(0.4)	8(0.5)	
	Daily	1(0.3)	3(0.2)	4(0.2)	
Fruits	Never	5(1.6)	38(2.5)	43(2.4)	0.22
	< 1 serving /day	112(35.7)	476(32.5)	588(33.1)	
	2–4 servings / day	167(53.5)	757(51.7)	924(52.0)	
	5–6 servings / day	16(5.1)	92(6.2)	108(6.1)	
	> 6 servings / day	13(4.1)	101(6.8)	114(6.4)	
Vegetables	Never	7(2.2)	33(2.2)	40(2.3)	0.30
	< 1 serving /day	90(28.7)	444(30.3)	534(30.1)	
	2–4 servings / day	186(59.6)	794(54.2)	980(55.1)	
	5–6 servings /day	17(5.4)	93(6.3)	110(6.2)	
	> 6 serving /day	13(4.1)	100(6.8)	113(6.4)	
Polished Rice	No	29(9.2)	109(7.4)	138(7.8)	0.28
	Yes	284(90.8)	1355(92.5)	1639(92.2)	
Palm oil for cooking	Sunflower	267(85.3%)	1186(81%)	1453(81.8%)	0.08
	Palm	25(8%)	183(12.5%)	208(11.7%)	
	Others ^a^	21(6.7%)	95(6.5%)	116(6.5%)	
Coffee	Never	224(71.3)	997(68.1)	1221(68.7)	0.47
	1–2 cups a day	86(27.70)	453(30.9)	539(30.3)	
	> 2 cups a day	3(1.0)	14(1.0)	17(1.0)	
Tea	Never	156(50.0)	760(51.9)	916(51.5)	0.80
	1–2 cups a day	145(46.2)	651(44.4)	796(44.8)	
	> 2 cups a day	12(3.8)	53(3.6)	65(3.7)	
Dairy	Never	51(16.6)	283(19.3)	334(18.8)	0.51
	< 1 per month	5(1.6)	18(1.2)	23(1.3)	
	1–3 times /month	0(0)	4(0)	4(0.2)	
	1–3 times/week	48(15.3)	181(12)	229(12.9)	
	> 3 times / week	2(0.6)	12(1)	14(0.8)	
	Daily	207(65.9)	966(66)	1173(66.0)	
Eggs	Never	74(23.9)	340(23)	414(23.3)	0.63
	< 1 per month	7(2.2)	34(2)	41(2.3)	
	1–3 times /month	16(5.1)	61(4)	77(4.3)	
	1–3 times/week	163(51.9)	816(56)	979(55.1)	
	> 3 times / week	17(5.4)	53(4)	70(3.9)	
	Daily	36(11.5)	160(11)	196(11.0)	
Chicken	Never	56(17.8)	282(19)	338(19.0)	0.89
	< 1 per month	6(1.9)	36(3)	42(2.4)	
	1–3 times /month	35(11.5)	142(10)	177(10.0)	
	1–3 times/week	198(63.1)	934(64)	1132(63.7)	
	> 3 times / week	18(5.7)	70(5)	88(5.0)	
Fish	Never	105(33.8)	570(39)	675(38.0)	0.34
	< 1 per month	25(8.0)	94(6)	119(6.7)	
	1–3 times /month	80(25.5)	383(26)	463(26.1)	
	1–3 times/week	103(32.8)	417(28)	520(29.3)	

Row percentages are included

* Statistically significant at 5% level of significance

^a^ others include, safflower oil, gingerly and rice bran oil

Table 3 provides the results of unadjusted and adjusted estimates in relation to GDM. The results reveal that eating red meat more than thrice a week is statistically significant (aRR = 2.1; (95% CI: 1.5,2.9) even after adjusting for other factors namely age, family history of diabetes mellitus, socioeconomic class, body mass index (BMI), the sum of skinfold thickness, metabolic equivalent of task (MET) and gestational age.

**Table 3:** Poisson regression model of effect of dietary habits on GDM status of pregnant women adjusted for confounders (*N* = 1777)

Variable	Categories	Unadjusted RR (95% CI)	***P***- value	Adjusted RR (95% CI)	***P***-value
**Red Meat consumption**	Never	1		1	
	< 1 per month	1.0(0.4,2.3)	0.98	1.2(0.6,2.4)	0.85
	1 or 3 times /month	0.8(0.49,1.2)	0.26	0.9(0.6,1.3)	0.41
	1–3 times/week	1.3(0.95,1.6)	0.11	1.3(0.98,1.6)	0.10
	> 3 times /week	2.6(1.8,3.8)	< 0.001*	2.1(1.5,2.9)	< 0.001*

Adjusted for age, family history of diabetes mellitus, socioeconomic class, *BMI* (body mass index), sum of skin fold thickness, *MET* (metabolic equivalent of task) and gestational age

1: reference category, *CI* Confidence Interval

* Statistically significant at 5% level of significance

## Discussion

To our knowledge, this is the first prospective study to assess the association between food habits during pregnancy and the risk of GDM among women attending public hospitals in India. In the absence of data from India, we, for the first time, show evidence that the use of red meat is significantly associated with GDM, similar to studies in different parts of the world [29, 37–39].

The meat consumption in India is on the rise, albeit much lower compared to other Western countries. We report in this study that red meat consumption and gestational diabetes are associated with relatively younger, mostly nulliparous women in the MAASTHI cohort, who are from the lower socioeconomic strata. Although red meat has emerged as a risk factor for colorectal cancer [40] and lung cancer [41] there are no studies in India to show the association between GDM and red meat consumption. Several causal mechanisms can explain GDM and red meat consumption. First, studies have shown that heme iron in red meat may be causing GDM [42], excess iron has been implicated in increasing the insulin resistance and the risk of type 2 diabetes. It is important to consider here that all public hospitals in India supplement pregnant women with Iron tablets irrespective of their iron status. Increased iron supplements in pregnant women without iron deficiency were related to an increased risk of GDM [43]. Thus, high red meat consumption and indiscriminate iron supplementation may be related to GDM. Qiet al earlier reported a higher consumption of red meat to increased risk of coronary heart disease among women with type 2 diabetes. Secondly, red meat is also high in saturated fat and cholesterol, thus affecting insulin sensitivity and beta-cell function [44, 45]. We found that there is inadequate consumption of vegetables and fruits in pregnant women attending public hospitals; women in lower socioeconomic strata often have limited access to nutritious food; their dietary choices are determined by affordable food items, social rank within the household and intrahousehold food allocation [46, 47]. According to national family health survey, more than half (50.4%) of pregnant women are anemic in India [48] and the recent comprehensive national nutrition survey revealed 30.9% of adolescents are vitamin B12 deficient [49]. On the other hand, rapid urbanization has resulted in nutrition transition, and it is estimated that 166 million adults are overweight and obese, and 73 million are diabetic [9]. Thus, India is at a unique juncture where both undernutrition and overweight has to be addressed by the public health system as non-communicable diseases are no longer the disease of the upper strata.

Among the strengths, this research was done in a fairly large sample with due considerations of confounding factors. Our data provide an independent risk factor for gestational diabetes that suggests that high red meat consumption thereof was associated with an increased risk of having GDM. These findings may be useful in dietary counselling during pregnancy. The limitations of the study are that we have not assessed portion size, and second, the questionnaires were not validated, and responses were recorded based on the participants’ recall. Third, we have not been able to adjust for dietary energy intake, we agree that it is a major limitation given that it might confound the overall association. Fourth, although we have identified associations between GDM incidence and red meat consumption, we do not suggest any causation and future studies are required to investigate this further.

## Conclusion

The results reported in this study indicated that a high intake of red meat is associated with an increased risk of having GDM. A further detailed evaluation of the diet, analyzing quality and quantity, is needed to understand the risk profile in this population better. Diet counselling for pregnant women at public hospitals can focus on preventing adverse pregnancy outcomes. Interventions to educate women in lower socioeconomic strata on inexpensive, seasonal, and healthy food might be helpful.

## Supplementary information

**Additional file 1.** Questionnaire. The file has the food habit questionnaire used in the study.

## Data Availability

The datasets used and analysed during the current study are available from the corresponding author on reasonable request.

## Abbreviations

BMI: Body mass index
GDM: Gestational diabetes mellitus
MET: Metabolic equivalent of task
T2DM: Type 2 diabetes mellitus
