# Supplementary material for: Food habits in pregnancy and its association with gestational diabetes mellitus: results from a prospective cohort study in public hospitals of urban India
Source: BMC Nutr. 2020 Nov 19;6:63. doi: 10.1186/s40795-020-00388-x (PMC7677816; doi:10.1186/s40795-020-00388-x)
Supplement: Supplementary file 1 — Additional file 1. Questionnaire. The file has the food habit questionnaire used in the study. [file 40795_2020_388_MOESM1_ESM.docx]

**Additional file 1**

Food Habit Questionnaire

1. How often do you have dairy products?

- ____ times/week
- ____ times/month
- Less than once per month
- Never
- Don’t Know
- Daily

1. How often do you have Eggs?

- ____ times/week
- ____ times/month
- Less than once per month
- Never
- Don’t Know
- Daily

1. How often do you have chicken?

- ____ times/week
- ____ times/month
- Less than once per month
- Never
- Don’t Know
- Daily

1. How often do you have fish?

- ____ times/week
- ____ times/month
- Less than once per month
- Never
- Don’t Know
- Daily

1. How often do you have red meats (mutton, pork, beef, etc.)?

- ____ times/week
- ____ times/month
- Less than once per month
- Never
- Don’t Know
- Daily

1. Do you regularly remove fat/skin from meat before cooking? :
2. Cooking oil you use most :

Sunflower oil

Safflower oil

Groundnut oil

Gingelly oil

Palm oil

Rice bran oil

Mustard oil

Coconut oil

1. Cereal you eat most :

Rice

Ragi

Wheat

Jowar

Maize

1. Do you use polished rice? :
2. Type of milk you consume most :

Whole milk

Skimmed milk

Toned milk

Skimmed milk powder

1. Do you use coconut as thickening agent? :
2. Do you use groundnut as thickening agent? : No
3. Do you use roasted bengal gram as thickening agent? :
4. Cooking method you use often :

Pressure Cooker

Frying

Cooking with the lid open

Cooking with the lid closed

Prolonged cooking

1. How often do you eat fried food? :

Daily,

About 4 to 6 times a week,

About 1 to 3 times a week,

Less than once a week,

Rarely,

Never

1. How often do you eat fresh fruits?

More than 6 servings per day,

About 5 to 6 servings per day,

About 2 to 4 servings per day,

Less than 1 serving per day,

Rarely,

Never

1. How often do you eat fresh vegetables?

More than 6 servings per day,

About 5 to 6 servings per day,

About 2 to 4 servings per day,

Less than 1 serving per day,

Rarely,

Never

1. How often do you eat outside? :

About 4 to 6 times a week,

About 1 to 3 times a week,

Less than once a week,

Rarely,

Never

1. Cups of coffee you consume daily :…………………………..
2. Cups of tea you consume daily :…………………………..
3. Do you discard excess water after cooking vegetables? :
4. Do you eat breakfast? :
5. How many coconuts do you use monthly? :…………………………..
6. Do you consume any nutritional supplements such as Horlicks, Complan, protein powder, etc.? :
7. Supplements you consume: ……………………………………………………………………
